# Local Enrichment of Multi-SNP Markers Improves Genomic Prediction from Low-Density Genotyping in Pacific White Shrimp

**DOI:** 10.3390/ijms27146202

**Published:** 2026-07-11

**Authors:** Tianzan Lyu, Ping Dai, Min Zhang, Maocang Yan, Guangfeng Qiang, Qiang Fu, Kun Luo, Xianhong Meng, Baolong Chen, Juan Sui, Xupeng Li, Junyu Liu, Mianyu Liu, Jian Tan, Jiawang Cao, Jie Kong, Hao Zhou, Sheng Luan

**Affiliations:** 1 State Key Laboratory of Mariculture Biobreeding and Sustainable Goods, Yellow Sea Fisheries Research Institute, Chinese Academy of Fishery Sciences, Qingdao 266071, China; lvtianzan@163.com (T.L.); daiping54@163.com (P.D.); qianggf1991@163.com (G.Q.); fuqiang@ysfri.ac.cn (Q.F.); luokun@ysfri.ac.cn (K.L.); mengxianhong@ysfri.ac.cn (X.M.); chenbl@ysfri.ac.cn (B.C.); suijuan@ysfri.ac.cn (J.S.); lixupeng@ysfri.ac.cn (X.L.); liujy@ysfri.ac.cn (J.L.); 2022213007@stu.njau.edu.cn (M.L.); tannjian@163.com (J.T.); caojw@ysfri.ac.cn (J.C.); kongjie@ysfri.ac.cn (J.K.); 2 Chinese Academy of Agricultural Sciences, Beijing 100081, China; 3 Zhejiang Mariculture Research Institute, Wenzhou 325005, China; milyzhang84@hotmail.com (M.Z.); yanmaocang@126.com (M.Y.)

**Keywords:** *Penaeus vannamei*, genomic selection, mSNP, genotype imputation, prediction accuracy

## Abstract

Low-density SNP panels are widely used to reduce genotyping costs in genomic selection (GS) in aquaculture, but sparse marker density can constrain prediction accuracy, particularly in species with rapid linkage disequilibrium decay. In this study, we evaluated a locally enriched multi-SNP (mSNP) strategy derived from a conventional 1K SNP panel in a family-based breeding population of Pacific white shrimp (*Penaeus vannamei*). Targeted sequencing was used to recover multiple nearby variants surrounding each marker locus, and conventional SNP and mSNP panels were compared for genomic features, imputation performance, and genomic prediction accuracy for body weight. The mSNP panel exhibited higher polymorphism and broader gene-region coverage than the corresponding SNP panel. The original mSNP panel improved prediction accuracy by 11.6% over the original 1K SNP panel. This advantage was maintained or slightly improved when mSNPs were restricted to on-target variants within ±300 bp of the target markers. A locus-matched comparison further showed that enriched mSNPs consistently outperformed conventional SNPs derived from the same target loci, with a 12.0% improvement at the full 961-locus panel. Although imputation improved the 1K SNP panel, the original mSNP panel already outperformed the imputed SNP panel and reached 0.509 after imputation. These findings demonstrate that locally enriched mSNP markers provide an effective and practical strategy for improving low-density GS in aquaculture species with rapid LD decay.

## 1. Introduction

Genomic selection (GS) enables early and accurate prediction of breeding values and has become an effective strategy for accelerating genetic improvement in aquaculture species [1,2,3,4]. Compared with pedigree-based selection, GS can better capture realized genetic relationships and Mendelian sampling, which is particularly valuable for traits that are costly, destructive, or difficult to measure at scale [5]. However, the routine implementation of GS in aquaculture breeding programs remains constrained by the cost of genotyping large numbers of selection candidates, especially in species with large family sizes and high selection intensity [6].

*Penaeus vannamei* is the most widely farmed shrimp species worldwide and a major target of selective breeding programs. These programs are typically characterized by large full-sib family sizes and strong family structure, which support high selection intensity but also substantially increase genotyping demand [7]. Low-density SNP panels have therefore been widely adopted as a practical approach to reducing genotyping costs. However, very low-density panels, such as 1K SNP chips, often capture limited genomic information, which can restrict genomic prediction accuracy. Genotype imputation provides one possible solution by inferring high-density genotypes from low-density data, but this strategy requires a high-density-genotyped reference population and therefore introduces additional cost and operational complexity [8,9,10]. Moreover, the effectiveness of imputation depends strongly on linkage disequilibrium (LD), reference population design, pedigree structure and marker density [11]. In species such as *P. vannamei*, where LD decays extremely rapidly, sparse SNP panels may have limited ability to tag nearby causal variants or shared chromosome segments, thereby limiting the benefit of imputation-based genomic prediction.

An alternative strategy is to increase the information captured at each targeted locus rather than relying solely on increasing marker density through imputation. Advances in targeted sequencing and liquid-phase capture technologies make it possible to recover multiple local SNPs surrounding a single target locus within a low-density genotyping framework [12]. The multi-SNP (mSNP) strategy can increase local allelic information per target locus without fundamentally changing the low-density genotyping framework [13]. Conceptually, unlike phased microhaplotypes, the mSNPs evaluated here were encoded as sets of locally recovered SNPs [14]. This distinction is particularly relevant in species with very rapid LD decay, where single-SNP markers may fail to maintain sufficient association with causal variation. Simulation studies in aquaculture have suggested that local multi-variant or microhaplotype-like marker systems can outperform equally sized SNP panels for genomic prediction and imputation. However, empirical evidence from real breeding populations remains limited [14].

In this study, we performed an empirical evaluation of an mSNP-based strategy for low-cost GS in *P. vannamei* using a real family-based breeding population. Here, mSNPs were defined as variants recovered by targeted sequencing around the 1K chip loci. Variants located within ±300 bp of the target loci were further classified as on-target mSNPs, whereas the remaining variants were classified as off-target mSNPs. We compared a conventional 1K SNP panel with the corresponding mSNP marker panel in terms of genomic coverage, polymorphism content and genomic prediction performance. Genotype imputation from low-density to high-density panels was also evaluated as a benchmark for assessing whether the mSNP strategy could outperform or complement the conventional low-density SNP plus imputation approach. By further separating on-target mSNPs from off-target variants, we tested whether the advantage of mSNPs was mainly attributable to enriched local allelic information around target loci. Together, this study provides empirical evidence that improving per-target marker informativeness can be a practical and cost-effective strategy for enhancing genomic prediction in shrimp breeding programs characterized by rapid LD decay.

## 2. Results

### 2.1. Pedigree Reconstruction, Phenotypic Summary, and Genetic Parameters

Comparison between COLONY-inferred assignments and the known family identity of the target individuals showed a concordance rate of 100%, indicating high accuracy of pedigree reconstruction. The reconstructed pedigree, comprising 50 full-sib families with 32 to 41 individuals per family, was used for subsequent pedigree-based analyses.

Descriptive statistics and genetic parameter estimates for body weight (BW) are summarized in Table 1. The mean BW of the communally reared G07 population was 10.88 ± 2.27 g, with individual values ranging from 3.30 to 18.70 g. Family mean BW ranged from 8.03 to 13.82 g, indicating substantial phenotypic variation both within and among families.

Based on the pedigree-based animal model, the additive genetic variance and residual variance for BW were estimated as 2.43 ± 0.54 and 2.50 ± 0.30, respectively, corresponding to a heritability of 0.49 ± 0.08. These estimates indicate that BW showed substantial additive genetic variation for subsequent genomic prediction analyses.

### 2.2. Comparative Genomic Features of SNP and mSNP Panels

After filtering, the 1K panel retained 961 SNP loci distributed across 43 chromosomes, with a maximum inter-marker gap of 2.53 Mb. The corresponding 1K mSNP panel retained 46,290 variants, reflecting the expansion of each target locus into multiple nearby variants and thereby providing substantially higher local marker density.

**Figure 1:**
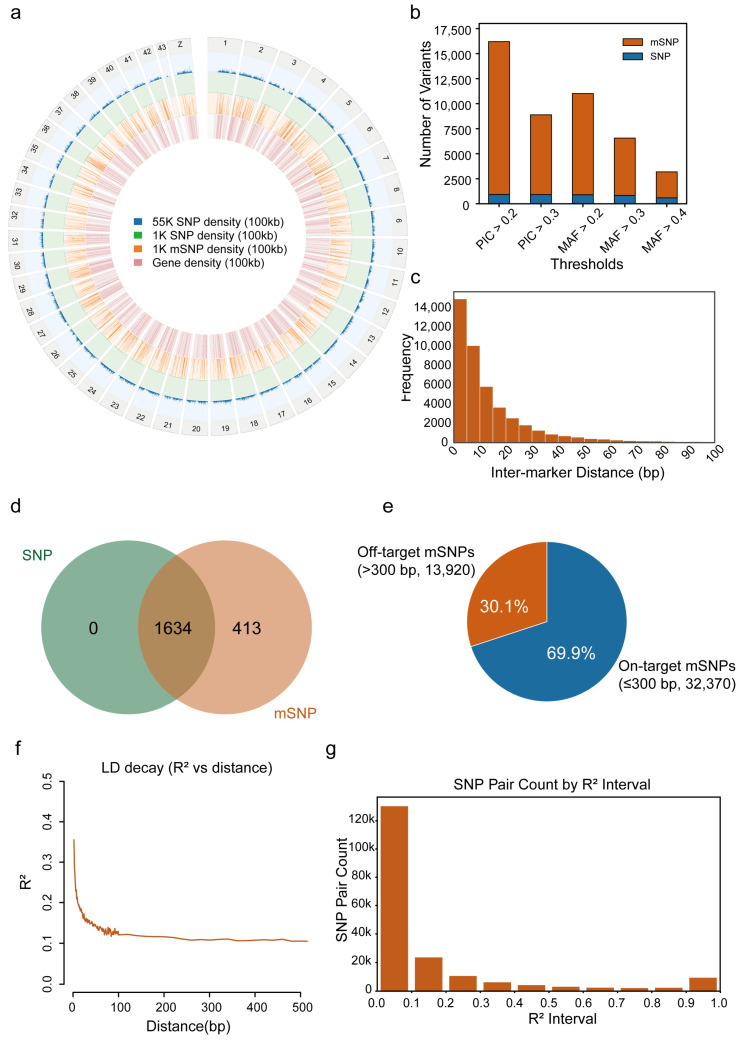
Comparative genomic feature analysis of the 1K SNP and mSNP panels. (**a**) Circos plot showing genome-wide distributions of 55K SNP density, 1K SNP density, 1K mSNP density, and gene density; colors correspond to the data categories indicated in the panel legend. (**b**) Variant counts under different polymorphism thresholds (PIC and MAF). (**c**) Frequency distribution of inter-marker distances for mSNP. (**d**) Overlap of gene-region annotations between SNP and mSNP. (**e**) Proportion of on-target and off-target mSNPs relative to the 1K target SNP loci, where on-target variants were defined as those located within ±300 bp of the target loci. (**f**) linkage disequilibrium (LD) decay with physical distance. (**g**) Distribution of LD strength (R^2^) across marker pairs.

Compared with the retained SNP panel, the mSNP panel extended genomic representation to 44 chromosomes, including chr43, which was not represented by the retained SNP loci (Figure 1a). mSNPs also showed substantially higher variant counts than SNPs under higher polymorphism information content (PIC) and minor allele frequency (MAF) thresholds (Figure 1b). In addition, mSNP density tended to be higher in gene-rich regions, and mSNPs covered all gene regions identified by SNPs while additionally annotating 413 unique gene regions (Figure 1a,d).

Inter-marker-distance analysis showed that more than 93.2% of mSNPs were separated by less than 60 bp, consistent with their local origin around targeted loci (Figure 1c). Among the 46,290 retained mSNPs, 32,370 (69.9%) were located within ±300 bp of the 1K SNP loci and were classified as on-target mSNPs, whereas the remainder were classified as off-target mSNPs (Figure 1e). To further evaluate whether the ±300 bp on-target boundary was appropriate, we conducted a sensitivity analysis using alternative local windows of ±100 bp, ±300 bp, and ±500 bp around the original 1K target SNPs. The number of retained on-target mSNPs increased from 13,203 within ±100 bp to 32,370 within ±300 bp and 38,127 within ±500 bp, corresponding to 28.5%, 69.9%, and 82.4% of all retained mSNPs, respectively. Thus, the ±100 bp window captured only a limited proportion of local variants, whereas the ±300 bp window retained the majority of mSNPs. Expanding the window from ±300 bp to ±500 bp provided only a modest additional gain in marker number. However, median MAF and PIC gradually decreased as the window expanded, from 0.094 and 0.156 at ±100 bp to 0.083 and 0.141 at ±300 bp and 0.079 and 0.135 at ±500 bp, respectively. These results indicate that the ±300 bp window provided a practical balance between retaining sufficient local marker information and avoiding excessive inclusion of more weakly informative variants.

LD analysis indicated generally weak linkage disequilibrium in the population. Overall, 79.2% of marker pairs showed R^2^ < 0.2, and LD decayed rapidly with physical distance, dropping below 0.2 within approximately 10 bp and stabilizing at around 0.14 after approximately 50 bp (Figure 1f,g).

### 2.3. Imputation Accuracy in SNP and mSNP Panels

**Figure 2:**
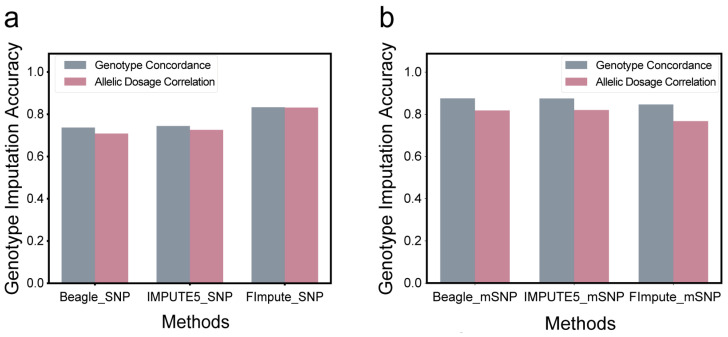
Genotype imputation performance for SNP and mSNP panels. (**a**,**b**) Genotype imputation performance for SNP (**a**) and mSNP (**b**) panels, evaluated by genotype concordance and allelic dosage correlation (r) using Beagle, IMPUTE5, and FImpute.

Imputation performance differed markedly between SNP and mSNP panels. For the SNP data (Figure 2a), FImpute showed the strongest performance, yielding a genotype concordance of 0.83 and an allelic dosage correlation of 0.83, both higher than those obtained with Beagle (0.74 and 0.71, respectively) and IMPUTE5 (0.74 and 0.73, respectively).

In contrast, for the mSNP panel (Figure 2b), Beagle and IMPUTE5 outperformed FImpute. Beagle achieved a genotype concordance of 0.87 and a dosage correlation of 0.82, whereas IMPUTE5 yielded corresponding values of 0.88 and 0.82; both exceeded those of FImpute (0.85 and 0.77, respectively). Thus, the ranking of imputation methods differed between SNP and mSNP panels, providing a basis for evaluating how imputation translated into downstream genomic-prediction performance.

### 2.4. Genomic Prediction Performance of Original SNP and mSNP Panels

**Figure 3:**
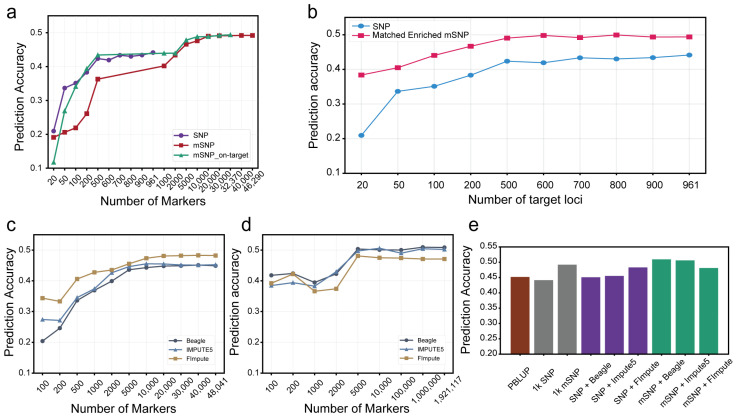
Genomic prediction performance of SNP, mSNP and locus-matched enriched mSNP panels. (**a**) Genomic prediction accuracy before imputation across marker densities for SNPs, mSNPs, and on-target mSNPs (±300 bp). (**b**) Genomic prediction performance between conventional SNPs and enriched mSNPs derived from the same target loci. For each target-locus density, the SNP panel and the enriched mSNP panel were constructed from identical genomic target regions. (**c**,**d**) Genomic prediction accuracy after imputation across marker densities for SNPs (**c**) and mSNPs (**d**), comparing Beagle, IMPUTE5, and FImpute. (**e**) Maximum genomic prediction accuracy across original and imputed panels, summarizing peak performance for SNP and mSNP panels under each imputation method.

Before evaluating the effect of imputation, genomic prediction performance was first assessed using the original 1K SNP and mSNP panels, with pedigree-based best linear unbiased prediction (PBLUP) included as a baseline reference. PBLUP yielded a prediction accuracy of 0.452 ± 0.038. Using the original marker datasets, genomic prediction performance differed substantially between SNP and mSNP panels (Figure 3a; Table 2). At extremely sparse densities, mSNP-based prediction was slightly lower than SNP-based prediction (0.191 vs. 0.210 at 20 markers). However, as marker density increased, mSNP-based prediction surpassed SNP-based prediction and reached a higher accuracy plateau.

The maximum prediction accuracy of the original SNP panel was 0.441 at 961 markers. In comparison, the mSNP panel reached 0.476 at 10,000 markers and plateaued at approximately 0.490–0.492 at higher densities, with a maximum value of 0.492. Thus, the maximum accuracy of the original mSNP panel was 11.6% higher than that of the original SNP panel. The original 1K SNP panel performed slightly below the PBLUP baseline, whereas the original mSNP panel exceeded it at moderate to high marker densities.

Restricting the analysis to the mSNPs within ±300 bp of the target loci did not reduce prediction performance and in some cases slightly improved it. For example, at 10,000 markers, prediction accuracy increased from 0.476 for the full mSNP panel to 0.488 for the on-target subset. The highest on-target accuracy reached 0.494 at 32,370 markers, remaining slightly above the peak value of the full mSNP panel (0.492). The predictive advantage of mSNPs therefore remained evident even when the analysis was restricted to target-proximal variants recovered from the local regions surrounding the original 1K target loci.

### 2.5. Locus-Matched Comparison Between SNPs and Locally Enriched mSNPs

To further distinguish the contribution of local marker enrichment from differences in genomic target selection, we performed a locus-matched comparison between conventional SNPs and enriched mSNPs derived from identical 1K target loci (Figure 3b). Across all evaluated target-locus densities, enriched mSNPs consistently achieved higher prediction accuracy than the corresponding SNP panels. The advantage was most pronounced at low marker density, where prediction accuracy increased from 0.210 to 0.384 at 20 loci, corresponding to an 82.9% relative improvement. As marker density increased, the relative gain gradually decreased but remained evident, reaching 12.0% at the full 961-locus panel, where enriched mSNPs achieved a prediction accuracy of 0.494 compared with 0.441 for the corresponding SNP panel. These results indicate that enriching local variants around predefined target loci improves genomic prediction beyond conventional single-SNP representation. Nevertheless, because enriched mSNP panels necessarily contained more variants per target locus than conventional SNP panels, this comparison does not completely separate local enrichment from increased regional marker density. Rather, by matching the target loci between panels, the analysis shows that the improvement was achieved through enriched local marker representation around the same genomic targets, rather than through selection of different target regions.

### 2.6. Effect of Imputation on Genomic Prediction Performance

Relative to the original panels described above, imputation improved genomic prediction in both marker systems, although the magnitude of improvement differed markedly between SNP and mSNP panels (Figure 3c–e; Table 3 and Table 4). Within the SNP panel, imputation increased prediction accuracy across marker densities, with FImpute providing the largest gains. At the highest density (48,041 SNPs), prediction accuracy increased from 0.441 before imputation to 0.482 after imputation with FImpute, corresponding to an improvement of 9.3%.

In contrast, gains from imputation were smaller for mSNP-based prediction. Using Beagle, the maximum prediction accuracy increased from 0.492 for the original mSNP panel to 0.509 after imputation, corresponding to a relative gain of 3.5%. IMPUTE5 yielded a similar maximum value of 0.504. Even without imputation, the mSNP panel consistently outperformed both the original SNP panel and the PBLUP baseline.

Overall, the gain from imputation was larger for the SNP panel than for the mSNP panel. FImpute yielded the largest improvement for the SNP panel, whereas Beagle and IMPUTE5 achieved the highest post-imputation prediction accuracies for the mSNP panel.

## 3. Discussion

This study provides the first empirical demonstration in *P. vannamei* that locally enriched mSNPs can improve low-density genomic prediction by increasing the information captured around each assayed locus. The central finding is not simply that more genome-wide markers improve prediction, but that local enrichment around selected low-density target loci provides a more informative marker representation than conventional single-SNP panel. At the full-panel level, the original mSNP panel outperformed the original 1K SNP panel before genotype imputation, indicating that its principal advantage did not depend on post hoc genotype reconstruction. This advantage was maintained when the analysis was restricted to on-target variants within ±300 bp of the original target loci, suggesting that the gain was mainly associated with local target-region information rather than incidental off-target variants. The locus-matched analysis further supports this interpretation. Because conventional SNPs and enriched mSNPs were derived from the same target loci, the observed advantage of enriched mSNPs cannot be explained simply by selecting different or more favorable genomic regions. Nevertheless, enriched mSNP panels also contained more local variants than the corresponding conventional SNP panels. Therefore, the observed improvement should be interpreted as the combined effect of local variant enrichment, increased regional marker density, and improved representation of local haplotypic or microhaplotype-like information, rather than as the effect of target-locus selection alone.

Although imputation improved prediction in both marker systems, its contribution was smaller than that of marker representation itself, especially for mSNPs. Together, these findings suggest that improving local marker representation may be more effective than relying primarily on sparse SNP panels followed by imputation in shrimp breeding populations characterized by rapid LD decay [15]. The exceptionally rapid LD decay observed in this population provides an important context for interpreting the advantage of mSNPs. When LD declines below R^2^ = 0.2 within approximately 10 bp, a single target SNP may have limited ability to tag nearby causal variants unless it is itself causal or extremely close to the causal polymorphism. By incorporating multiple local variants around the same target locus, mSNPs may increase the probability of capturing variants that are tightly linked to causal loci. In addition, multiple adjacent or nearby variants may jointly represent local haplotype or microhaplotype-like information, which can provide more informative regional genetic signals than a single SNP. From the perspective of GBLUP, enriched local marker representation may also improve the ability of the genomic relationship matrix to capture realized relatedness and Mendelian sampling differences among related individuals in a family-based population. Therefore, the improved prediction accuracy of mSNPs likely reflects a combination of enhanced marker–QTL associations and improved relationship estimation.

The definition of on-target mSNPs also requires careful interpretation. In this study, variants within ±300 bp of the original target SNPs were considered on-target variants because this interval represented the major local enrichment region around target loci and was consistent with the expected range of the target-enrichment design. The sensitivity analysis using alternative local windows further suggests that the choice of window size involves a balance between local information recovery and signal dilution. A very narrow window may preserve high physical proximity to the target SNP but exclude many locally recovered variants, thereby reducing the multi-variant representation of each target locus. In contrast, an overly broad window may capture more variants but can also introduce distant or weakly informative markers that dilute the local signal. The ±300 bp window therefore represents a practical operational compromise for defining target-proximal mSNPs. Given the rapid LD decay in this population, this boundary should not be interpreted as a strict LD-based interval, but rather as a local regional enrichment boundary reflecting targeted capture and local marker aggregation. Future studies should further evaluate optimal window sizes under different LD structures and target-enrichment designs.

The comparative genomic features of the two marker systems support this interpretation. The mSNP strategy expanded each targeted locus into multiple nearby variants, resulting in higher polymorphism content and broader gene-region coverage than the corresponding SNP panel. Importantly, the mSNP panel covered all gene regions represented by the SNP panel and additionally annotated 413 unique gene regions. These genomic characteristics suggest that mSNPs increased local variant density around selected loci while simultaneously expanding functional gene-region coverage. The on-target analysis further strengthened this conclusion by showing that variants located within ±300 bp of the original panel loci were sufficient to maintain the predictive advantage of mSNPs. Therefore, the improvement was mainly attributable to local marker representation around target loci rather than to the incidental inclusion of off-target variants.

This interpretation is consistent with previous studies on multi-variant marker systems. Guo, et al. [12] proposed that capturing multiple nearby SNPs per target locus can increase marker informativeness at similar cost, whereas Delomas, et al. [14] showed in simulated oyster and salmon breeding programs that microhaplotype-based panels could achieve higher imputation and breeding-value accuracy than equally sized SNP panels. For example, 150 microhaplotype loci in Pacific oyster yielded similar breeding-value accuracy to approximately 350 greedy-selected SNPs, representing a potential panel-size reduction of roughly 57%. However, evidence from real breeding populations remains limited. Our study extends these observations by providing direct empirical validation in a real shrimp breeding population and, importantly, by separating the effects of local marker representation from those of post hoc imputation.

The advantage of mSNPs is biologically plausible in *P. vannamei*, a species characterized by very rapid short-range LD decay. Under such conditions, a single sparse SNP may have limited ability to remain associated with nearby causal variation or with short chromosome segments shared among relatives, especially when marker density is constrained by genotyping cost [16,17]. By recovering multiple nearby variants around each target locus, the mSNP strategy increases the probability that at least part of the local marker signal remains informative for quantitative trait loci or realized genetic relationships [14,18]. Under rapid LD decay, locally enriched mSNPs may partially compensate for the limited tagging ability of sparse single-SNP markers by increasing local redundancy of marker–QTL associations. From the perspective of GBLUP, enriched local marker representation should improve the ability of the genomic relationship matrix to capture both realized relatedness and local genetic signals linked to causal variations [19]. This mechanism plausibly explains why the mSNP panel reached a higher prediction plateau than the conventional SNP panel.

The imputation results further clarify the relative roles of marker design and genotype reconstruction. For the original SNP panel, imputation produced a clear increase in prediction accuracy, with FImpute performing best, which is consistent with the strong family structure of the study population [20]. In contrast, the gain from imputation was smaller for the mSNP panel, although Beagle and IMPUTE5 performed well for the denser mSNP-derived datasets. This contrast is informative: the sparse SNP panel was more strongly limited by missing local information, so imputation could recover a larger proportion of useful signal, whereas the original mSNP panel had already captured much of the local variation relevant to genomic prediction. The different ranking of imputation methods is also consistent with their underlying assumptions. FImpute tends to perform well in low-density datasets with strong family structure, whereas Beagle and IMPUTE5 are better able to exploit denser local haplotypic information [21,22]. Similar patterns have been reported by Kriaridou, et al. [23], who found that FImpute generally performed best across several aquaculture species, but also identified Pacific oyster as an exception, where imputed low-density panels underperformed the original unimputed panels, likely because of rapid LD decay. The comparable pattern observed in *P. vannamei* suggests that imputation is most effective when local LD is sufficient to support accurate genotype inference, whereas in species with rapid LD decay, improving the original marker representation may be the more effective lever for increasing prediction accuracy.

These findings have direct implications for practical genomic selection in shrimp breeding. In family-based breeding programs, selection candidates are typically close relatives of the reference population, and the prediction setting evaluated here broadly reflects this deployment scenario. Within this context, the advantage of the mSNP strategy is both predictive and operational. Under the pricing scheme used in this study, the direct genotyping cost of the mSNP strategy for the present panel was estimated at 82,680 CNY, calculated as (1923 + 144) × 40 CNY. In contrast, the conventional 1K SNP plus imputation strategy was estimated at 94,200 CNY, calculated as 1923 × 40 CNY + 144 × 120 CNY. Thus, the mSNP strategy reduced direct genotyping cost by approximately 12.2% while achieving prediction accuracy comparable to, or slightly higher than, that of the imputed SNP strategy. More broadly, the present results move the question from conceptual plausibility to empirical validation by showing, in a real breeding population, that improving local marker representation may provide a more effective route for low-cost genomic prediction than relying primarily on sparse SNP panels followed by imputation in species with rapid LD decay [6,24].

Several limitations should be noted. First, the present evaluation was based on a single family-based breeding population and focused on body weight; therefore, the generality of the results across additional traits, generations, and breeding populations remains to be further validated. Second, although the cross-validation design was appropriate for evaluating genomic prediction within the studied population, prediction accuracy may partly reflect the close genetic relationships among individuals and the capture of realized genomic relationships among relatives. Consequently, the performance of mSNP panels across generations, across genetically distant breeding lines, or in populations with weaker relatedness may be lower than that observed in the present study. Independent-generation and multi-population validation will therefore be important for assessing the transferability of mSNP-based prediction. Third, mSNPs were treated here as locally enriched SNP sets rather than phased multiallelic haplotypes. Future studies could compare SNP-set, haplotype, and microhaplotype encoding strategies to determine how best to represent local variant information. Finally, the cost advantage of the mSNP strategy may vary depending on sequencing platform, panel design, and population scale. Despite these limitations, the consistent advantage of enriched mSNPs across marker densities suggests that local marker enrichment is a promising strategy for improving low-density genomic prediction in Pacific white shrimp breeding populations.

## 4. Materials and Methods

### 4.1. Sample Collection

**Figure 4:**
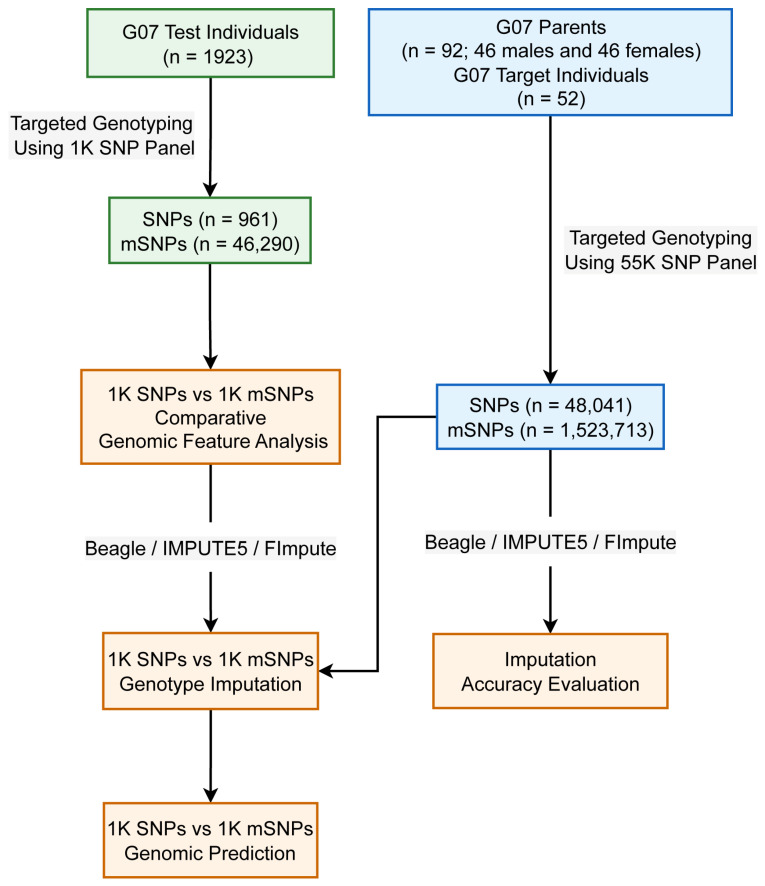
Experimental design and analytical workflow of the study.

An overview of the experimental design and analytical workflow is presented in Figure 4. The study population was derived from a nucleus breeding population of *P. vannamei* established in 2017, corresponding to the G07 generation of a selective breeding program maintained by Zhejiang Mariculture Research Institute. A total of 50 full-sib families were included, comprising 1923 individuals for phenotypic and genetic analyses, with family sizes ranging from approximately 32 to 41 individuals (mean ≈ 38). All individuals were treated as a single breeding population in subsequent analyses.

All families were reared under standardized indoor culture conditions in two cement tanks to minimize environmental heterogeneity. Juveniles with an initial mean body weight (IBW) of 0.071 g were stocked at a density of 66.67 individuals m^−2^ and reared for 116 days. The mean IBW of each full-sib family was recorded and included as a fixed covariate in the genetic evaluation model. Seawater salinity was maintained at 20 ± 2 throughout the culture period under routine management. At harvest, individuals were measured for BW and sampled for genotyping.

To facilitate pedigree reconstruction using the 1K SNP panel, a subset of individuals was selected as target individuals. A total of 52 target individuals from separately maintained families were used as reference genotypes for identifying the family origin of communally reared test individuals and for subsequent parentage and sibship inference.

### 4.2. Genotyping and Quality Control

Muscle tissue from 1923 G07 offspring was genotyped using the “Yellow Sea Chip No. 1” low-density panel (1K) [25] by MolBreeding Biotechnology Co., Ltd. (Shijiazhuang, China). In addition, 144 individuals were genotyped using the corresponding high-density array (55K) [25], including 92 parents (46 males and 46 females), and 52 target individuals from the G07 generation. These high-density genotypes were used in the subsequent imputation analyses.

Genomic DNA was extracted from muscle tissue using the GenoPrep Polyphenol DNA Extraction Kit (MolBreeding Biotechnology Co., Ltd., Shijiazhuang, China; magnetic bead-based method), and DNA quality and concentration were assessed using the DropAnalyzer Plus workstation (MolBreeding Biotechnology Co., Ltd., Shijiazhuang, China). Target capture libraries were prepared using GenoBaits probe-capture technology (MolBreeding Biotechnology Co., Ltd., Shijiazhuang, China) and sequenced on the DNBSEQ-T7 platform (MGI Tech Co., Ltd., Shenzhen, China).

Clean reads were aligned to the reference genome using BWA v0.7.17 [26] with default parameters, and variant calling was performed using SaiLe Caller v1.0.3.6. Variant-level filtering was conducted using GATK v4.3 [27] with the following thresholds: QD < 2.0, MQ < 40.0, FS > 60.0, SOR > 3.0, MQRankSum < −12.5, and ReadPosRankSum < −8.0. Additional quality control was performed using PLINK v2.0 [28] with criteria of minor allele frequency (MAF) ≥ 0.01, genotype missing rate ≤ 0.10, and variant quality (QUAL) ≥ 200.

After filtering, 46,290 variants constituting the mSNP panel were retained. Based on the predefined 1K chip marker list, 961 SNP loci were extracted using BCFtools v1.17 [29] for downstream analyses.

### 4.3. Parentage and Sibship Assignment

To reconstruct pedigrees for downstream analyses, parentage and sibship assignment were performed using COLONY v2.0.7.0 [30] under a full-likelihood framework. Analyses were conducted using 961 high-quality SNPs selected from the 1K panel. The program was run under the assumptions of monogamy for both sexes, no inbreeding, and a weak sibship prior. Candidate parental genotypes, when available, were supplied with known sex information. The analysis was performed with medium run length and precision, and the genotyping error rate was set to 0.05. Assignments with confidence ≥ 95% were retained for downstream pedigree reconstruction.

Assignment was carried out in three steps. First, target individuals were analyzed to infer sibship structure, allowing COLONY to cluster individuals into full-sib families and reconstruct virtual sires and dams for each family when complete parental information was unavailable. Concordance between COLONY-inferred family assignments and the recorded family identity of the target individuals was used to assess assignment accuracy. Second, available parental genotypes were incorporated as candidate parents to replace virtual parents and validate the inferred family assignments. Third, all remaining offspring were assigned to the validated families using the target individuals and real parents as references. This procedure enabled reliable pedigree reconstruction even when parental genotypes were incomplete, and the reconstructed pedigree was subsequently used for relationship-matrix construction and downstream genetic analyses.

### 4.4. Comparative Genomic Feature Analysis of 1K SNP and mSNP Panels

Comparative genomic feature analyses were performed to evaluate the genomic distribution, annotation characteristics, and local LD properties of the SNP and mSNP panels. Genome-wide marker distributions across chromosomes were visualized using the R package Circlize v0.4.1 [31], and functional annotation was conducted using SnpEff v5.4 [32]. Additional figures were generated using custom Python scripts in Python v3.9.

For local classification, variants located within ±300 bp of each original 1K target SNP were defined as on-target mSNP variants, whereas variants outside this interval were defined as off-target variants. The ±300 bp window was used as an operational definition of target-proximal variants based on the targeted capture design and the empirical distribution of recovered variants, rather than as a strict LD-based boundary. This interval covered the majority of recovered local variants surrounding the assayed target loci and was used to distinguish variants likely recovered from the intended target-proximal region from more distant off-target variants. To evaluate the robustness of this operational boundary, a sensitivity analysis was further performed using alternative local windows of ±100 bp, ±300 bp, and ±500 bp around the original 1K target SNPs. For each window, the number and proportion of retained on-target mSNPs were calculated, and variant-level polymorphism and informativeness metrics, including MAF and PIC, were summarized. Genome coverage and marker density were calculated using VCFtools v0.1.16 [33], and LD decay was estimated using PopLDdecay v3.4 [34] based on pairwise R^2^ values.

### 4.5. Genotype Imputation for SNP and mSNP Panels

#### 4.5.1. Imputation Accuracy Evaluation

Imputation accuracy was evaluated using 52 target individuals as an independent validation set under two scenarios: (i) imputing from the 1K SNP panel to the high-density SNP panel (48,041 loci), and (ii) imputing from the 1K mSNP panel to the corresponding high-density mSNP panel (1,523,713 loci), which was constructed based on the 92-parent reference panel.

For each validation individual, high-density genotypes were masked to the corresponding low-density SNP or mSNP panel derived from the 1K target loci and then imputed back to high density. Imputation accuracy was assessed by direct comparison between the imputed genotypes and the original high-density genotypes using genotype concordance and allelic dosage correlation.

The reference panel used for imputation accuracy evaluation consisted only of the 92 parents genotyped at high density. Genotype imputation was performed using Beagle v5.4 [35], SHAPEIT5 [36] combined with IMPUTE5 [37], and FImpute v3.0 [38]. For Beagle and IMPUTE5, effective population size was set to 43 based on estimation using NeEstimator v2.0 [39]. Beagle was run with default settings for window size, overlap and iteration parameters unless otherwise specified. For the IMPUTE5 analysis, genotypes were first phased using SHAPEIT5 and then imputed with IMPUTE5 using the same effective population size. For FImpute, the pedigree information reconstructed by COLONY was incorporated to exploit family relationships during imputation. All three imputation methods were applied to the same masked low-density datasets and evaluated against the original high-density genotypes of the validation individuals to ensure a fair comparison.

#### 4.5.2. Imputation for Offspring

Following the imputation-accuracy evaluation, the same imputation strategy was applied to the full G07 offspring population. At this stage, the reference panel was constructed from the 92 parents and 52 target individuals genotyped at high density.

Offspring genotyped with the 1K SNP panel were imputed to the high-density SNP panel, and the corresponding low-density mSNP panel was imputed to the high-density mSNP panel using the same three methods described above. The resulting imputed datasets contained 48,041 SNPs and 1,921,117 mSNPs, respectively, and were used for subsequent genomic prediction analyses. The larger number of retained mSNP loci in this analysis reflects the expanded reference panel, which included both the 92 parents and the 52 target individuals.

### 4.6. Genomic Prediction Using Original and Imputed Marker Panels

Genetic evaluation was performed using the following linear mixed model:

(1) $$y_{ijk}=\mu +{Sex}_{j}+b_{1}{IBW}_{k}+a_{i}+e_{ijk}$$

where $y_{ijk}$ is the observed BW of the *i*th individual; μ is the overall mean; ${Sex}_{j}$ is the fixed effect of the *j*th sex (male or female); ${IBW}_{k}$ is included as a linear covariate with regression coefficient $b_{1}$; $a_{i}$ is the random additive genetic effect of the *i*th individual, and $e_{ijk}$ is the random residual effect, assumed to follow $e∼N\left(0,\;I\sigma_{e}^{2}\right)$, where $\sigma_{e}^{2}$ is the residual variance. In the PBLUP analysis, $a∼N\left(0,\;A\sigma_{a}^{2}\right)$, where A is the pedigree-based additive relationship matrix derived from the reconstructed pedigree and $\sigma_{a}^{2}$ is the additive genetic variance. PBLUP analyses were performed using ASReml-W v4.2 [40]. In the GBLUP analysis, a $∼N\left(0,\;G\sigma_{a}^{2}\right)$, where G is the genomic relationship matrix constructed using the VanRaden method [41]. GBLUP analyses were performed using HIBLUP v1.6 [42]. The same genomic prediction framework was applied to both the original and imputed SNP and mSNP panels.

To evaluate prediction accuracy, phenotypes were pre-adjusted using the pedigree-based model above, and adjusted phenotypes were obtained by removing the estimated fixed effects:

(2) $$y_{ijk}^{*}=y_{ijk}-{\hat{Sex}}_{j}-\hat{b_{1}}{IBW}_{k}$$

The adjusted phenotypes were used only for calculating prediction accuracy and were not used as input for breeding-value estimation. Fixed effects were estimated using the full dataset only to obtain adjusted phenotypes for evaluating prediction performance, whereas breeding-value estimation and validation were performed strictly within the cross-validation framework.

Prediction performance was evaluated across marker-density gradients. For each marker density level, five random marker subsets were generated, and each subset was evaluated using ten independent replicates of fivefold cross-validation. Prediction accuracy was defined as the mean Pearson correlation between adjusted phenotypes and predicted breeding values in the validation sets. The same cross-validation partitions were applied across prediction methods within each replicate. To further evaluate the contribution of local marker enrichment while controlling for genomic target selection, a locus-matched comparison was performed between conventional SNPs and enriched mSNPs derived from the same 1K target loci. For each target-locus density, the same set of 1K target loci was used to construct both marker panels. The conventional SNP panel retained only the original target SNP at each locus, whereas the enriched mSNP panel included the retained local mSNP variants recovered around the corresponding target loci. The same marker-sampling replicates and cross-validation partitions were applied to the paired SNP and enriched mSNP panels.

## 5. Conclusions

This study demonstrates that locally enriched mSNPs provide a more effective marker representation than conventional low-density SNP panels for genomic prediction in *P. vannamei*. By capturing multiple nearby variants around each target locus, the mSNP strategy increased local marker representation, expanded gene-region coverage, and achieved higher prediction accuracy than the original 1K SNP panel. The advantage was retained in the on-target mSNP subset, indicating that the improvement mainly resulted from local marker representation rather than incidental off-target variants. Although genotype imputation improved SNP-based prediction, the unimputed mSNP panel already outperformed the imputed SNP panel, and imputation further improved the performance of the mSNP panel. Together, these findings suggest that improving per-target marker informativeness through mSNP design is a practical and cost-effective strategy for enhancing genomic selection from low-density genotyping in shrimp breeding programs and other aquaculture species with rapid LD decay.

## Figures and Tables

**Table 1:** Descriptive statistics and genetic parameters for body weight.

Parameter	Value
Number of families	50
Total individuals	1923
Family size range	32–41
Mean BW	10.88 g ± 2.27 g
Range (min–max)	3.3–18.7 g
Phenotypic variance ($\sigma_{p}^{2}$)	4.92
Additive variance ($\sigma_{a}^{2}$)	2.43 ± 0.54
Residual variance ($\sigma_{e}^{2}$)	2.50 ± 0.30
Heritability (h^2^)	0.49 ± 0.08

**Table 2:** Genomic prediction accuracy of original marker panels.

Marker Density	SNP (Mean ± SD)	mSNP (Mean ± SD)	On-Target mSNP (Mean ± SD)
20	0.210 ± 0.038	0.191 ± 0.047	0.117 ± 0.038
50	0.337 ± 0.040	0.206 ± 0.044	0.269 ± 0.039
100	0.351 ± 0.042	0.219 ± 0.048	0.341 ± 0.040
200	0.383 ± 0.043	0.261 ± 0.053	0.394 ± 0.042
500	0.424 ± 0.044	0.363 ± 0.046	0.434 ± 0.036
600	0.419 ± 0.043	—	—
700	0.433 ± 0.040	—	—
800	0.430 ± 0.044	—	—
900	0.434 ± 0.044	—	—
961	0.441 ± 0.043	—	—
1000	—	0.402 ± 0.050	0.440 ± 0.035
2000	—	0.434 ± 0.045	0.440 ± 0.036
5000	—	0.466 ± 0.042	0.478 ± 0.035
10,000	—	0.476 ± 0.039	0.488 ± 0.037
20,000	—	0.490 ± 0.042	0.489 ± 0.038
30,000	—	0.491 ± 0.041	0.492 ± 0.040
32,370	—	—	0.494 ± 0.040
40,000	—	0.492 ± 0.041	—
46,290	—	0.492 ± 0.042	—

Note: “—” indicates that the corresponding marker density was not evaluated for that dataset.

**Table 3:** Genomic prediction accuracy of the imputed SNP panel.

Marker Density	Beagle (Mean ± SD)	IMPUTE5 (Mean ± SD)	FImpute (Mean ± SD)
100	0.204 ± 0.042	0.274 ± 0.040	0.344 ± 0.041
200	0.246 ± 0.042	0.271 ± 0.040	0.333 ± 0.049
500	0.336 ± 0.038	0.345 ± 0.037	0.406 ± 0.046
1000	0.369 ± 0.038	0.374 ± 0.041	0.428 ± 0.051
2000	0.399 ± 0.042	0.426 ± 0.038	0.435 ± 0.046
5000	0.436 ± 0.040	0.446 ± 0.039	0.455 ± 0.041
10,000	0.443 ± 0.042	0.455 ± 0.040	0.473 ± 0.040
20,000	0.448 ± 0.039	0.455 ± 0.038	0.481 ± 0.042
30,000	0.449 ± 0.040	0.452 ± 0.040	0.482 ± 0.042
40,000	0.451 ± 0.041	0.451 ± 0.040	0.483 ± 0.043
48,041	0.449 ± 0.042	0.452 ± 0.040	0.482 ± 0.043

**Table 4:** Genomic prediction accuracy of the imputed mSNP panel.

Marker Density	Beagle (Mean ± SD)	IMPUTE5 (Mean ± SD)	FImpute (Mean ± SD)
100	0.418 ± 0.039	0.385 ± 0.038	0.392 ± 0.034
200	0.424 ± 0.045	0.394 ± 0.042	0.422 ± 0.037
500	0.463 ± 0.040	0.460 ± 0.040	0.436 ± 0.041
1000	0.395 ± 0.045	0.382 ± 0.039	0.366 ± 0.042
2000	0.423 ± 0.042	0.430 ± 0.035	0.374 ± 0.044
5000	0.503 ± 0.036	0.497 ± 0.034	0.481 ± 0.040
10,000	0.501 ± 0.040	0.506 ± 0.035	0.475 ± 0.043
100,000	0.500 ± 0.039	0.490 ± 0.039	0.474 ± 0.040
1,000,000	0.509 ± 0.038	0.504 ± 0.037	0.471 ± 0.039
1,921,117	0.509 ± 0.038	0.502 ± 0.037	0.471 ± 0.039

## Data Availability

The data presented in this study are only available on request from the corresponding author due to the protection of breeding population information.
